# The effects of metformin and alendronate in attenuating bone loss and improving glucose metabolism in diabetes mellitus mice

**DOI:** 10.18632/aging.203729

**Published:** 2022-01-14

**Authors:** Qiyun Zhou, Zhiqiang Guan, Shengfu Liu, Yanjiao Xuan, Gang Han, Hua Chen, Xiao Jin, Kun Tao, Zhiyuan Guan

**Affiliations:** 1 Department of Orthopedics, The Shanghai Tenth People’s Hospital of Tongji University, Shanghai, PR China; 2 Department of Orthopedics, Qinghai Provincial People’s Hospital, Xining, Qinghai, PR China; 3 Department of Dermatology, The First People’s Hospital of Xuzhou, Xuzhou, Jiangsu 221002, PR China; 4 Department of Radiology, Beijing Jishuitan Hospital, Beijing 10035, PR China; 5 Department of Rheumatology and Immunology, The First People’s Hospital of Xuzhou, Xuzhou, Jiangsu 221002, PR China

**Keywords:** alendronate, metformin, diabetes osteoporosis, combined medication

## Abstract

Background: To explore the anti-osteoporosis and anti-diabetes effects and potential underlying mechanisms of treatment with metformin and alendronate in diabetes mellitus mice.

Methods: Eight-week-old C57 BL/KS db/db and db/+ female mice were evaluated according to the following treatment group for 12 weeks: control group, diabetes mellitus group, diabetes mellitus with metformin group, diabetes mellitus with Alendronate group, diabetes mellitus with metformin plus alendronate group. Glucose level, glucose tolerance test, bone mineral density, bone microarchitecture, bone histomorphometry, serum biomarkers, and qPCR analysis.

Results: Combined metformin and alendronate can improve progression in glucose metabolism and bone metabolism, including blood glucose levels, blood glucose levels after 4 and 16 hours fasting, glucose tolerance test results, insulin sensitivity and reduces bone loss than the diabetes group. The use of alendronate alone can increase significantly serum glucagon-like peptide-1 levels than the diabetes group. The use of metformin alone can improve bone microstructure such as Tb.Sp and Tb.N of spine in diabetic mice.

Conclusion: The combined use of alendronate and metformin has an anti-diabetes and anti-osteoporotic effect compared with diabetic mice, but they appear to act no obvious synergistically between alendronate and metformin.

## INTRODUCTION

Globally, diabetic patients have quadrupled in the past 30 years and diabetes has become the ninth major cause of death. One in eleven adults worldwide suffers from diabetes and 90% of diabetes patients suffer from type 2 diabetes [1, 2]. Osteoporosis is also one of the diseases that seriously impair the health of the elderly. In the United States, osteoporosis contributed about 1.5 million fractures each year [3, 4]. Osteoporosis and diabetes mellitus are both chronic diseases and show a clear link to morbidity and mortality [5]. Complications of diabetes were also significantly associated with fractures after falling [6–9].

Metformin, an anti-diabetic drug, can significantly improve the progression of a variety of diseases, including polycystic ovary syndrome, tuberculosis, cardiovascular [10], and neurological diseases [11]. The effects of metformin on bone metabolism have also been studied [12, 13]. Existing research recognizes that Metformin played a critical role in osteoporosis by inhibiting systemic inflammation and promoting osteoclast formation [14–16]. Anti-osteoporosis drugs such as alendronate can also improve the progression of diabetes. In the clinical study, administration of 70 mg/week alendronate improves fasting plasma glucose, HbA1c, and insulin indices in postmenopausal women [17]. However, anti-osteoporotic treatment does not alter the development of diabetes in the meta-analysis [18].

Because diabetes and osteoporosis are both important metabolic diseases, it is very important to know whether the combined use of anti-osteoporosis drugs and anti-diabetic drugs will improve the progression of diabetes and the progression of diabetic osteoporosis. Therefore, the purpose of our study consisted of analyzing the effects of combined metformin and alendronate on diabetic-induced bone loss and possible mechanisms.

## MATERIALS AND METHODS

### Animal experimental procedures

Eight-week-old C57 BL/KS db/db female mice (Lepr-KO/KO, *n* = 36) and non-diabetic (C57BLKS-Lepr-db/+, *n* = 9) were purchased from Beijing Vital River Laboratory Animal Technology (Beijing, China). A blood glucose level higher than 11.1 mmol/L was considered a hyperglycemic state. All experimental protocols and animal handling procedures were conducted according to the recommendations in the Guide for the Care and Use of Laboratory Animals, published by the National Institutes of Health (Publications No. 80-23, revised in 1996). This study was approved by the Experimental Animal Committee of our hospital.

After one week of adaptive feeding (mice were maintained in a standard animal facility with controlled temperature (22°C) and photoperiod (12 h light and 12 h dark) and free access to freshwater and food), mice were raised in the SPF animal house after treatment. and then random grouping was carried out after the treatment, divided into five groups, such as control group (Con, C57BLKS-Lepr-db/+, *N* = 9), diabetes mellitus group (DM, *N* = 9), DM with metformin group receiving daily metformin at 113.75mg/kg (DM + MET, *N* = 9) [19], Alendronate group receiving daily alendronate at 0.5 mg/kg (DM + ALE, *N* = 9) [20], metformin plus Alendronate group receiving daily alendronate at 0.5 mg/kg and metformin at 113.75 mg/kg (DM + MET + ALE, *N* = 9). The Control group was treated with no metformin or alendronate at all. All drugs were administered intragastrically in normal saline solution beginning at 8 weeks of age and lasting for 12 weeks.

### Fasting blood glucose (FBG) and oral glucose tolerance test (GTT)

For the fasting blood glucose, glucose tolerance assays, and insulin tolerance exam as described by Amir [21]. After 12 weeks of treatment, fasted mice (16 h, paper bedding) by monitoring glucose levels after a glucose bolus (1 g/kg of body weight (BW)) or insulin (0.5 U/kg BW) by intraperitoneal (IP) injection. The exam of glucose was carried from fasted (4–16 h) or re-fed animals (15 min to 1 h). Re-feeding was conducted by injecting a bolus of glucose (1 g/kg of BW) IP as mentioned above. The first drop of blood was thrown away and then the second drop of blood was detected by the glucometer (Roche Diagnostics, Mannheim, Germany). We collected the tail blood samples at 0, 15, 30, 60, and 120 min after glucose loading and detected the blood glucose value by the glucometer (Roche Diagnostics, Mannheim, Germany).

### Serum markers examination

Serum OCN, GLP-1, CTX-1, and TRAP 5b concentrations (*n* = 9 for each group) of all mice in the current study were quantified after fasting for 8 h using commercial enzyme-linked immunosorbent assay (ELISA) kits (CUSABIO Biotech Co., Wuhan, China) according to the manufacturer’s instructions.

### Radiology examination

BMD of the whole tibiae and L4 were measured by a DXA (Faxitron^®^ LX-60 Cabinet radiography system, US) and Micro-CT (Inveon, Siemens, Erlangen, Germany). The scanning parameters used were 70 kVp, 111 μA, and 1000 projections per 180°, resulting in a 10.5 μm isotropic voxel size and a total scan time of 13.2 min. Trabecular bone micro-architecture was assessed using the μCT Evaluation Program (Image Processing Language v. 5.0A, Scanco) [22, 23].

### Biomechanical examination

The collected femurs were wrapped up using etamine soaked with normal saline, which was then stored at low temperatures. The length of the specimen was the full length of the femur. Specimens were subjected to compressive loading on a biomechanical testing machine with a loading speed of 2 mm/min [20].

### Histomorphometry

The proximal tibia was dehydrated, embedded, sliced and then the calcein double-labeling sections were analyzed, which included mineral apposition rate (MAR) and bone formation rate per bone surface (BFR/BS) [24].

### qPCR

Tibias were cleaned of muscle and connective tissue, flash-frozen in liquid nitrogen, and stored at −80°C. Frozen tibias were crushed under liquid nitrogen conditions with a Bessman tissue pulverizer (Spectrum Laboratories, Rancho Dominguez, CA, USA). Total RNA was extracted using Trizol reagent (Invitrogen, Carlsbad, CA, USA). The expression levels of bone metabolism, glucose metabolism, and inflammation-related genes, including ALP, OCN, BMP-2, Runx-2, Beclin-1, AMPK, OPG, COL1A1, Gfi1, OPN, GPR43, GPR41, GCG, RANKL, PC1/3, which has been upload in Supplementary Table 1. The relative change in gene expression was analyzed by the 2^−ΔΔCT^ method. The mRNA of ALP, OCN, BMP-2, Runx-2, Beclin-1, AMPK, OPG, COL1A1, Gfi1, OPN, RANKL, is calculated with the DM group as the baseline, and the mRNA of GPR43, GPR41, GCG, PC1/3 is calculated with the control group as the baseline.

### Statistical analysis

All measurements are presented as the mean ± standard deviation (SD) and a *P*-value of ≤0.05 was considered statistically significant. Bodyweight of the time-course study was analyzed by two-way repeated-measures analysis of variance (ANOVA). Data were analyzed for intervention and time main effects. The data were analyzed using GraphPad Prism 8.02 (La Jolla California, USA) and one-way ANOVA followed by Tukey’s multiple.

### Ethical approval

All procedures performed in studies involving animal were in accordance with the ethical standards of the institutional (LA221221).

## RESULTS

### The effect of metformin and alendronate on body weight and food intake

**Figure 1:**
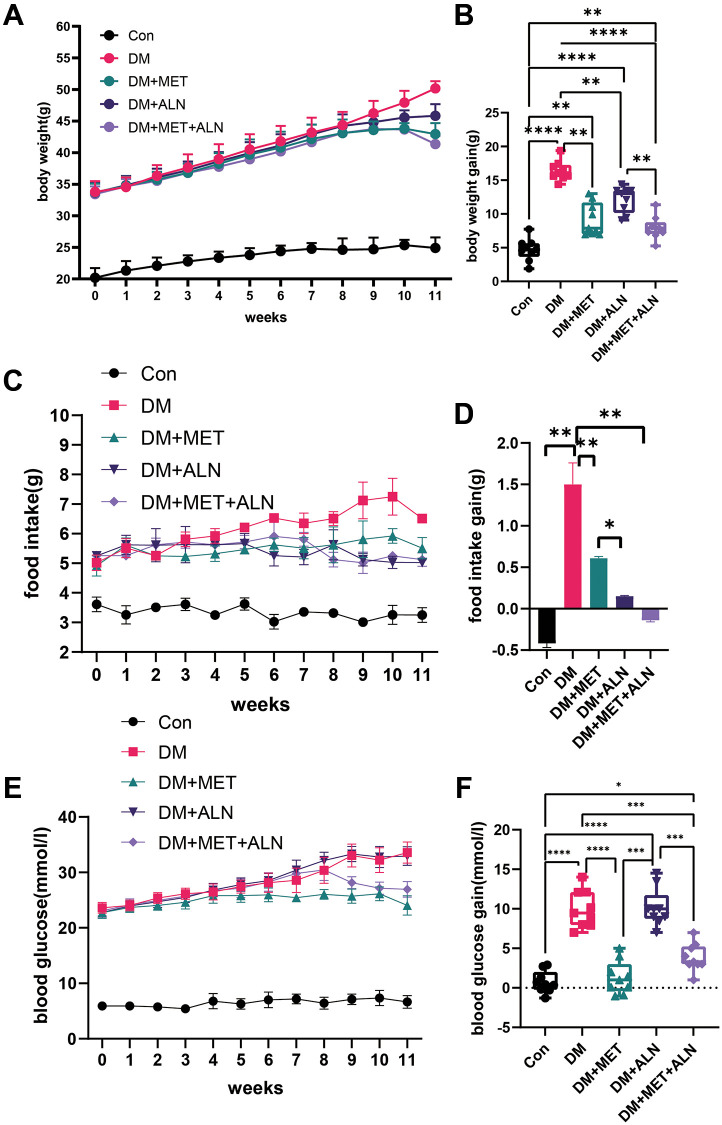
**Effect of body weight, food intake and blood glucose in metformin and alendronate in diabetes mice.** (**A**, **B**) Change of body weight in four group. (**C**, **D**) Change of food intake. (**E**, **F**) Change of blood glucose.

We analyzed the changes in the body weight and found that diabetic mice increased body weight significantly. After treatment with metformin and alendronate alone, the bodyweight significantly reduced compared with the diabetic group and the combined treatment also decreased significantly in the bodyweight than treatment with metformin and alendronate alone (Figure 1A, 1B). The food intake has also shown some kind of pattern with a change of body weight (Figure 1C, 1D).

### The effect of metformin and alendronate on glucose metabolism

**Figure 2:**
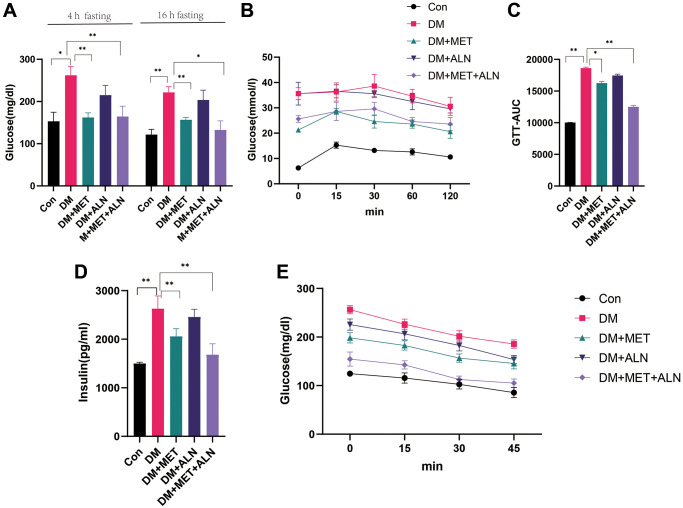
**Effect of glucose metabolism in metformin and alendronate in diabetes mice.** (**A**) serum glucose after 4 hour and 16 hours fasting. (**B**, **C**). GTT test. (**D**, **E**) Serum insulin and Insulin sensitivity.

Because metformin and alendronate also have significant effects on glucose metabolism, we assessed the effects of using alone or in combination alendronate and metformin treatment on glucose metabolism. We found that the use of metformin alone and the combined use of metformin and alendronate significantly improved blood glucose levels, but the use of alendronate alone did not improve blood glucose significantly (Figure 1E, 1F). The experiment of serum glucose test after 4 hour fasting and 16-hour fasting, GTT test and insulin levels and insulin sensitivity can only be improved in both the use of metformin alone and the combined use of metformin and alendronate (Figure 2A–2C) but the use of alendronate has a weaker effect on glucose metabolism (Figure 2D, 2E).

### The effect of metformin and alendronate on bone microstructure

**Figure 3:**
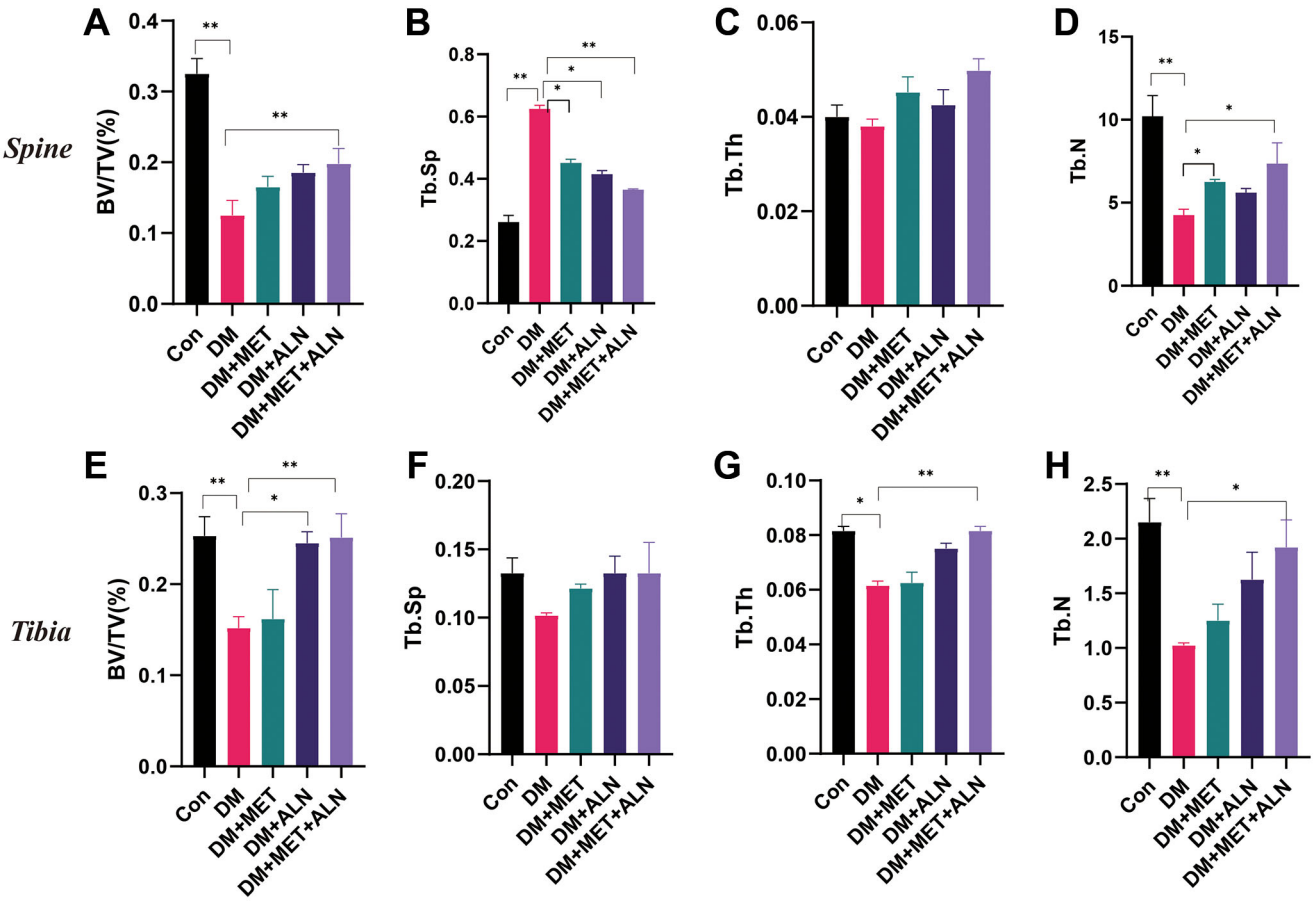
**The effect of metformin and alendronate on bone microstructure.** (**A**) BV/TV in spine. (**B**) Tb.Sp in spine. (**C**) Tb.Th in spine. (**D**) Tb.N in spine. (**E** ) BV/TV in tibia. (**F**) Tb.Sp in tibia. (**G**) Tb.Th in tibia. (**H**) Tb.N in tibia.

We found that combined utilization of alendronate and metformin can improve the BV/TV, Tb.N, Tb.Sp of the spine and BV/TV, Tb.Th and Tb.N of the tibia than diabetes group. besides, the use of alendronate alone can be increased significantly in BV/TV of tibia and decreased significantly in Tb.Sp of spine. The use of metformin alone can improve Tb.N and Tb.Sp of spine than diabetes mice (Figure 3A–3H).

**Figure 4:**
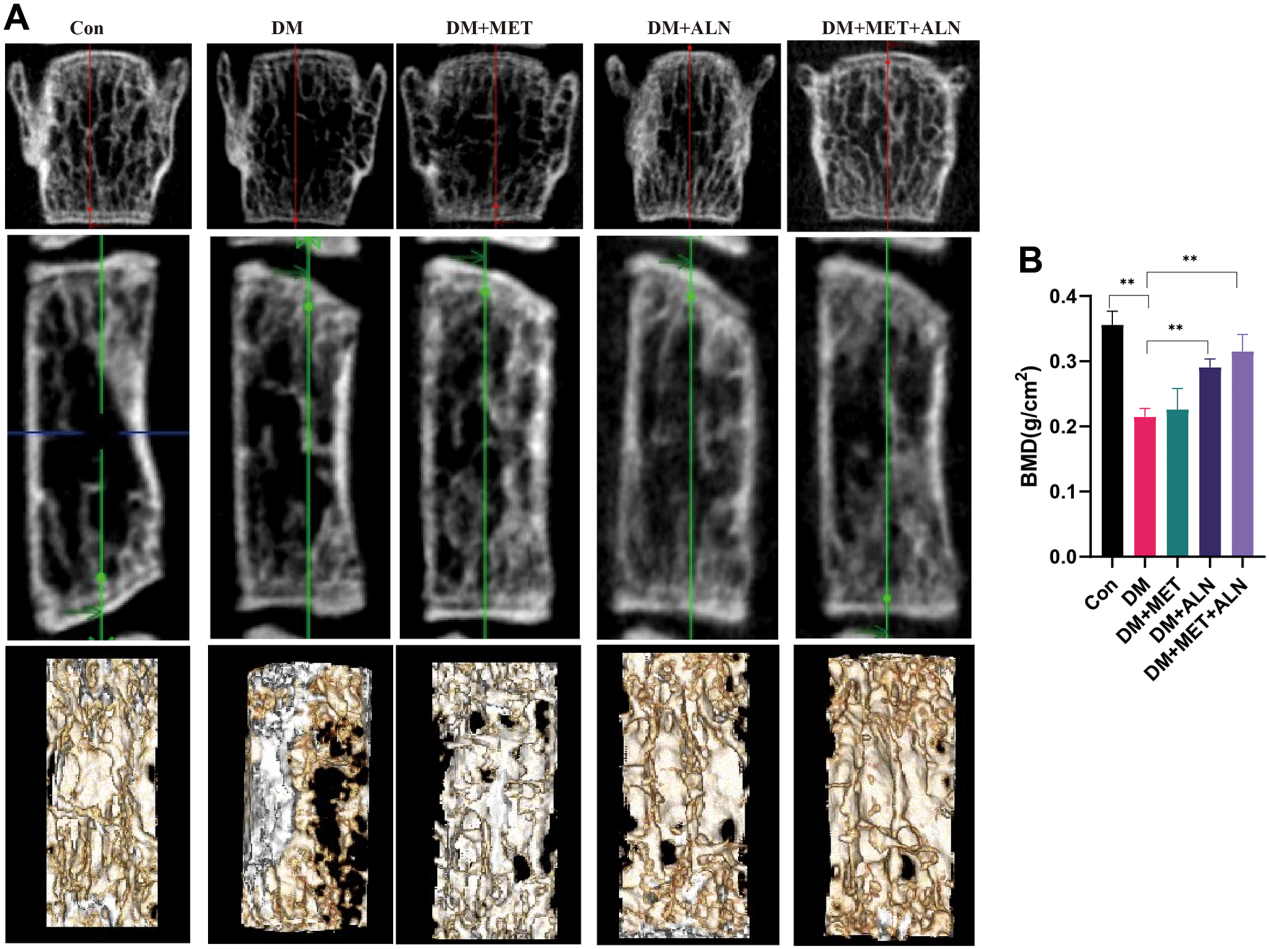
**The effect of metformin and alendronate on bone microstructure in lumbar vertebra.** (**A**) 3D image of Micro-CT. (**B**) BMD of spine.

**Figure 5:**
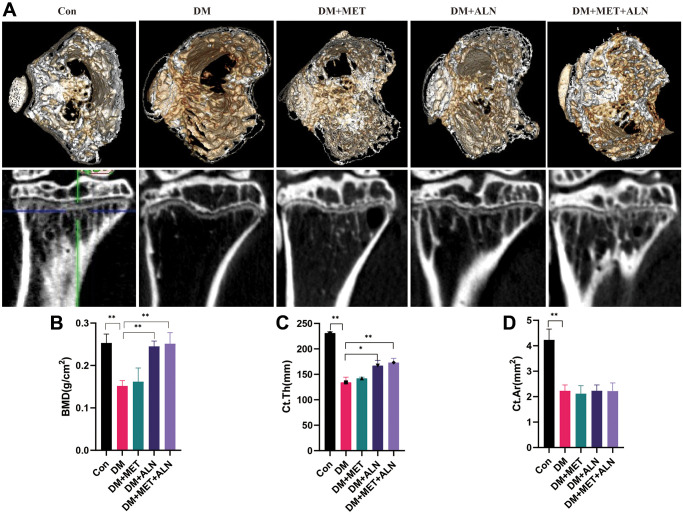
**The effect of metformin and alendronate on bone microstructure in tibia.** (**A**) 3D image of Micro-CT in tibia. (**B**) BMD of tibia. (**C**) Ct.Th in tibia. (**D**) Ct.Ar in tibia.

DXA analysis found that diabetes can decrease significantly in bone mass density (BMD) of the spine and tibia. The use of alendronate alone and the combined use of alendronate and metformin can significantly improve bone loss, but for metformin, it does not significantly improve BMD of the spine and tibia (Figure 4, Figure 5A, 5B).

For the results of tibial cortical bone, it was found that the use of alendronate alone and the combined use of alendronate and metformin can improve the thickness of cortical bone, but there is no significant effect on the area of cortical bone (Ct.Ar) (Figure 5C, 5D).

### The effect of metformin and alendronate on pathology and biomechanics

**Figure 6:**
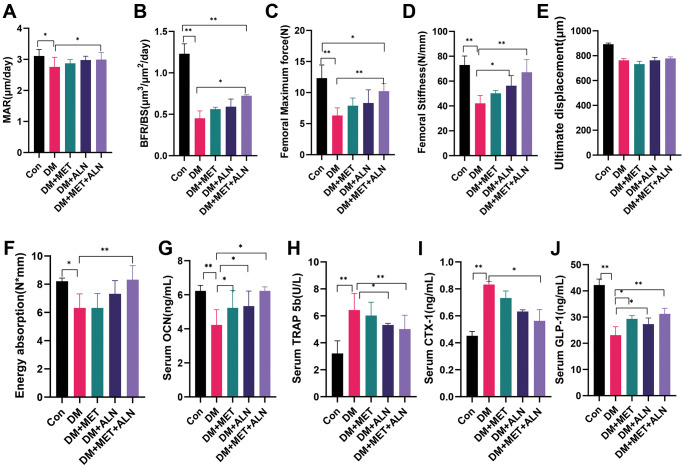
**The effect of metformin and alendronate on serum biomarker, pathology and biomechanics.** (**A**) mineral apposition rate (MAR). (**B**) bone formation rate per bone surface (BFR/BS). (**C**) maximum load. (**D**) stiffness. (**E**) ultimate displacement. (**F**) energy absorption. (**G**) serum OCN. (**H**) Serum TRAP 5b. (**I**) Serum CTX-1. (**J**) Serum GLP-1.

We found that the combination of alendronate and metformin can increase the result of MAR and BFR/BS than diabetic mice and shown that combined medications can promote bone formation. We also analyzed the biomechanics of the tibia and found that combination drugs significantly improved maximum force. Stiffness, and energy absorption but the use of metformin alone also had no significant effect on the maximum force (Figure 3, Figure 6A–6F).

### The effect of metformin and alendronate on serum biomarkers and mRNA level in the tibia

We further analyzed the results of serological markers and found that diabetic mice significantly reduced OCN and GLP-1 levels, but significantly increased TRAP 5b and CTX-1 levels. Combined medication can significantly reverse the process. In addition, the use of alendronate alone can significantly reduce TRAP 5b levels and increase GLP-1 and the use of metformin alone can significantly increase GLP-1 and OCN levels (Figure 6G–6J).

**Figure 7:**
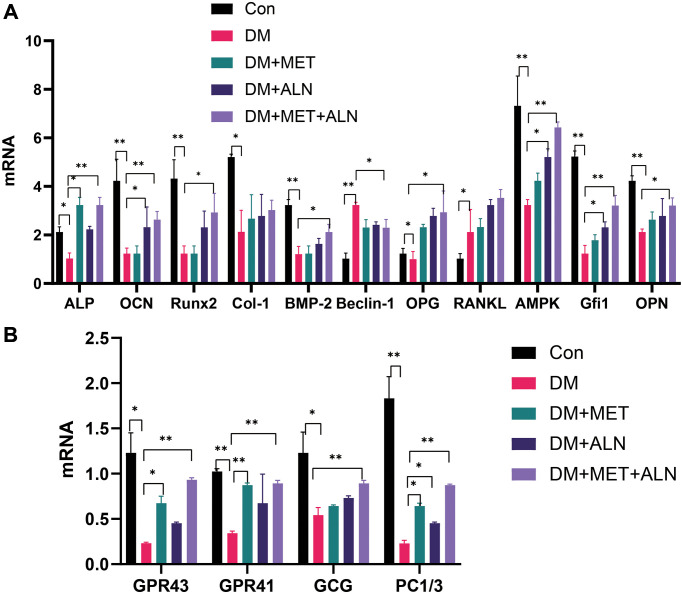
**The relative mRNA level of tibia.** (**A**) bone metabolism relative mRNA level. (**B**) glucose metabolism relative mRNA level.

We firstly analyzed the expression of bone metabolism-related genes in the tibia. Diabetic mice decrease significantly in ALP, OCN, Runx2, Col-1, BMP-2, OPG, AMPK, Gfi1, and OPN and decreased significantly in Beclin-1 than the control group (Figure 7A). Besides, the use of Metformin alone can only increase significantly in ALP and the use of alendronate alone can increase significantly in OCN, AMPK, Gfi1 mRNA level than the control group. Secondly, we further analyzed the level of mRNA related to glucose metabolism in the tibia and found that the combined use of metformin and alendronate can significantly increase the levels of GPR43 and GPR41, GCG, and PC1/3 than diabetic mice. The use of metformin alone can also increase significantly in GPR43 and GPR41 and PC1/3 than in diabetic mice (Figure 7B).

## DISCUSSION

In our research, the study found that the combined use of alendronate and metformin can substantially improve bone loss and glucose metabolism disorders than the diabetes group. However, the hypoglycemic effect or anti-osteoporosis effect of combining the use of alendronate and metformin is not obvious compared with the use of metformin alone or alendronate alone. It means that they appear to act no obvious synergistically between alendronate and metformin.

Osteoporosis is often accompanied by diabetes [25] and bone remodeling is compromised in both type 1 and 2 diabetes [26, 27]. Both body weight and food intake are significantly affected by diabetes and medication. Diabetes will enhance the weight of the patient and anti-diabetic treatment can improve the weight change of the patient [28–30]. This is also compatible with our research results. At the same time, alendronate can also substantially improve weight gain [31, 32]. The improvement in body weight and food intake may better reflect the results of the improvement in bone metabolism and glucose metabolism [33–36].

The treatment of diabetes with anti-osteoporosis drugs has drawn attention [37, 38]. In our studies, we found that the use of alendronate alone can decrease significantly in 4- and 16-hour fasting glucose and increased GLP-1 level but has not significantly decreased glucose level and insulin sensitivity in diabetic mice. It is In accordance with the present results, previous studies (e.g. Konstantinos et al.) have demonstrated that alendronate was associated with a significant 50% reduction in the risk of incident T2DM [39]. Yang et al. also found that antiresorptive therapy was not a risk factor for diabetes [40]. Alendronate also play important role in improving fasting plasma glucose and insulin sensitivity and decreases insulin resistance in prediabetic osteopenic postmenopausal women [17]. However, in our animal experiments, we found that the use of alendronate alone has a weaker effect on glucose metabolism than the use of metformin which can improve blood glucose and insulin sensitivity.

At the same time, we have also observed that the use of metformin alone can improve the bone microstructure of the spine than the diabetes group. Consistent with the literature, this research found that participants who reported that metformin use is involved in a lower risk of osteoporosis in adult women independent of type 2 diabetes mellitus and obesity [11, 41]. This also accords with our earlier observations, which showed that the metformin decreased bone turnover marker and influence bone formation in clinical studies [42, 43]. However, we have also noticed that the effect of metformin on bone metabolism is significantly weaker than the use of alendronate, so the use of metformin alone in the treatment of diabetic osteoporosis may require further verification. In addition, contrary to expectations, this study did not find a significant difference between the anti-osteoporosis effect of using the alendronate alone or the anti-diabetes effect of use in the alendronate and combination medication. These results further support the idea that the synergy between metformin and alendronate may be poor. However, the levels observed in this investigation are far below those observed by Lyudmila et al. It is suggested that metformin combined with alendronate significantly reduced the degree of cartilage degeneration and show the synergy in osteoarthritis. In addition, metformin can reverse some of the complications caused by alendronate, such as stomach damage [44]. A probable explanation for these results may be the lack of adequate observation time. Therefore, in our future research, we will further increase the observation time to determine whether the combined use of metformin and alendronate has a synergistic effect.

Metformin increased ALP and OCN secretion, enhanced BMP-2 expression, improved bone mineral density (BMD) [45]. In our study, it was found that metformin can improve the secretion of OCN, but not significant improvements in osteoclast-related biological markers (for example, CTX-1 and TRAP 5b), which may indicate that metformin may play important role in the process of osteoblast [46]. Metformin can also improve the progression of diabetes through the AMPK regulation and affect the expression of BMP-2 levels, and it can also improve bone metabolism [47, 48]. AMPK-Gfi1-OPN axis also play important role in bone and glucose metabolism [49]. Mai et al. found that metformin stimulates OPN and reduces RANKL expression in osteoblasts and ovariectomized rats [50–52]. OPN mediated AMPK regulation of osteogenesis and inhibited adipogenesis [49]. Besides, metformin also can inhibit the gene level of Runx2 which is associated with osteoblast differentiation markers such as OCN [53].

The effect and related mechanism of bisphosphonate on osteoporosis treatment has been widely studied [54–57]. Alendronate has a certain effect on the development of diabetes [58, 59]. Ikeda et al. found that alendronate that produces a reduction in urinary NTx and inhibition of decrease in BMD may have a clinical significance to reduce the risk of bone fracture in postmenopausal type 2 diabetic women [60]. In this study, we found that alendronate can increase the secretion of GPL-1 and proconvertase 1/3 (PC1/3) activity which may improve glucose metabolism [61, 62].

In our study, it was further verified that combination applications had significant improvements in diabetic osteoporosis, but the use of metformin or bisphosphonates alone did not improve osteoporosis or diabetes significantly, which also indicated that single medication in diabetic osteoporosis treatment may not be effective and further study in the clinical practical application of combination drugs.

## CONCLUSIONS

In conclusion, the present study demonstrated that combining metformin and alendronate prevent the emergence of diabetes and diabetic-relative bone loss by up-regulating AMPK gene expression and secreting GLP-1, stimulating bone formation and suppressing bone resorption than diabetes group. But they appear to act no obvious synergistically between alendronate and metformin in a shorter observation period. Future studies on the current topic are therefore recommended to focus on the effect of combining the use of alendronate and metformin in diabetes and diabetic-relative bone loss.

## Supplementary Materials

Supplementary Table 1
