# Supplementary material for: The effects of metformin and alendronate in attenuating bone loss and improving glucose metabolism in diabetes mellitus mice
Source: Aging (Albany NY). 2022 Jan 14;14(1):272–85. doi: 10.18632/aging.203729 (PMC8791222; doi:10.18632/aging.203729)
Supplement: Supplementary Table 1 [file aging-14-203729-s001.pdf]

SUPPLEMENTARY TABLE

Supplementary Table 1. Primer sequences used for RT-QPCR.

	Forward primer sequences	Reverse primer sequences
GAPDH	AACTTTGGCATTGTGGAAGG	ACACATTGGGGGTAGGAACA
GPR43	CTGGCACAGTTCCTTGATCCTCAC	GGCAGCAGCAGCAACAGGAG
GPR41	TGCTCATCTTCTTCGTCTGCTTCG	GTCGGCTTGGAACTTGGAGGAAG
GCG	ATTCATTGCTTGGCTGGTGA	CCAGAATGGTGCTCATCTCG
PC1/3	ACATGGGGAGAGAATCCTGTAGGCA	CATGGCCTTTGAAGGAGTTCCTTGT
ALP	TGGTTACTGCTGATCATTCCCACG	AATGTAGTTCTGCTCATGGACGCC
COL1A1	GCTCCTCTTAGGGGCCACT	ATTGGGGACCCTTAGGCCAT
OCN	AAGCAGGAGGGCAATAAGGT	TAGGCGGTCTTCAAGCCAT
BMP-2	TATGGATCCACTTAAGATGG TGGCCGGGACCCGCTGT	TATTGCGGC CGCTTAACGACACCCGCAGCCCTC
Runx-2	CCTTCCAGACCAGCAGCAG	TCCGTCAGCGTCAACACCA
Beclin-1	GCTGTAGCCAGCCTCTGAAA	AATGGCTCCTGTGAGTTCCTG
AMPK	TGATGATGAGGCTGTGAA	TAGAGGCGAGG TAGAACT
OPG	GAGGAGTCTGGTAGTGGTTCC	GGGCGTTTCGTTGAATATGCG
Gfi1	5-TTCTCTCGCTGCGGAGTCT-3	AGGCACTAGAAATGACTTGAAAGAAAA
OPN	AGACACTTTCACTCCAATCGTCCC	TGGTTCATCCAGCTGACTTGACTC
RANKL	GCGCAACAGTGTTTCCACAG	CACGCTTGGATCACAGTAAGG
